# Supplementary material for: First-4-week erythrocyte sedimentation rate variability predicts erythrocyte sedimentation rate trajectories and clinical course among patients with pyogenic vertebral osteomyelitis
Source: PLoS One. 2019 Dec 4;14(12):e0225969. doi: 10.1371/journal.pone.0225969 (PMC6892503; doi:10.1371/journal.pone.0225969)
Supplement: S1 Table — (DOCX) [file pone.0225969.s001.docx]

**S1 Table.** Variability of erythrocyte sedimentation rate during the first 4 weeks following pyogenic vertebral osteomyelitis diagnosis (*N* = 342), based on treatment duration and recurrence status.

| **Variables**, median (IQR) | **Treatment Duration** | | |  | | **6-Month Recurrence** | | |
| --- | --- | --- | --- | --- | --- | --- | --- | --- |
|  | **<12 weeks (N = 172)** | **≥12 weeks (N = 170)** | ***P*-value^a^** |  | **No**  **(N = 280)** | | **Yes**  **(N = 42)** | ***P*-value^a^** |
| Initial ESR value, mm/h | 71.5 (48.8, 90) | 85.5 (66, 102) | < 0.001 |  | 78.5 (54, 95) | | 82 (67.2, 102) | 0.255 |
| ESR **–** AD^b^, mm/h | -9 (-33, 8) | -9 (-22, 8) | 0.395 |  | -9 (-28, 8.2) | | -11.5 (-29, 4) | 0.770 |
| ESR **–** CV^c^, % | 25.2 (14.8, 37.8) | 17.6 (7.6, 29) | < 0.001 |  | 21.1 (10.6, 35) | | 20.5 (11.1, 34.5) | 0.984 |
| ESR **–** PC^d^, % | -15 (-41.2, 11.6) | -10.8 (-28.1, 10.8) | 0.144 |  | -12.9 (-36.6, 12.1) | | -12.5 (-37.6, 6.3) | 0.895 |
| Intercept^e^, mm/h | 71.8 (56.9, 87.9) | 84.9 (68, 96.1) | < 0.001 |  | 79.6 (62.1, 93) | | 85.2 (66.4, 95.1) | 0.203 |
| Slope^e^, mm/h | -0.5 (-1, -0.1) | -0.4 (-0.8, 0) | 0.078 |  | -0.5 (-0.9, 0) | | -0.6 (-1, -0.2) | 0.363 |
| Days from initial to last  measure | 21 (15, 25) | 21 (14.2, 25) | 0.939 |  | 21 (14.8, 25) | | 21 (14.2, 24.8) | 0.945 |

**Abbreviations:** AD, absolute difference; CV, coefficient of variation; ESR, erythrocyte sedimentation rate; IQR, interquartile range; PC, percent change; PVO, pyogenic vertebral osteomyelitis.

1. *P*-values are calculated by Wilcoxon rank-sum test.
2. Absolute difference between the first ESR value and the last within the first 4 weeks following PVO diagnosis.
3. Coefficient of variation = (Standard deviation/mean) x 100.
4. Percent change from the first to last ESR value during the first 4 weeks following PVO diagnosis x 100.
5. Intercept and slope were calculated using a multilevel model including both a random intercept and slope with all ESR measurements clustered within the patients.
