# Supplementary material for: First-4-week erythrocyte sedimentation rate variability predicts erythrocyte sedimentation rate trajectories and clinical course among patients with pyogenic vertebral osteomyelitis
Source: PLoS One. 2019 Dec 4;14(12):e0225969. doi: 10.1371/journal.pone.0225969 (PMC6892503; doi:10.1371/journal.pone.0225969)
Supplement: S2 Table — (DOCX) [file pone.0225969.s002.docx]

**S2 Table.** The 4-week erythrocyte sedimentation rate (ESR) variability and 6-month ESR trajectory by the surgical treatment.

| **Variables** | **Immediate operation**  **(N = 206)** | **Delayed operation**  **(N = 65)** | **Drainage without operation (N = 21)** | **No operation**  **(N = 109)** | **p-value** |
| --- | --- | --- | --- | --- | --- |
| **ESR variability within 4 weeks of PVO diagnosis, mean ± SD** |  |  |  |  |  |
| Initial ESR | 75.8 ± 29.5 ^b^ | 80.6 ± 26.8 ^b^ | 67.4 ± 18.3 | 74.5 ± 27.2 | 0.587 |
| ESR-AD | -9.3 ± 29.9 ^c^ | -4.4 ± 30.9 ^c^ | -14.1 ± 33.4 | -11.6 ± 25.9 | 0.489 |
| ESR-CV | 26.1 ± 18.1 | 20.8 ± 14.1 | 27.6 ± 20.9 | 23.2 ± 18.0 | 0.254 |
| ESR-PC | 9.5 ± 202.6 | 3.7 ± 49.2 | -13.0 ± 48.1 | -9.6 ± 49.0 | 0.315 |
| ESR-intercept | 76.3 ± 21.1 | 83.5 ± 20.3 | 69.8 ± 12.1 | 77.4 ± 20.3 | 0.845 |
| ESR-Slope | -0.4 ± 0.7 | -0.4 ± 0.7 | -0.7 ± 0.9 | -0.5 ± 0.6 | 0.220 |
| **ESR trajectory within 6 months of PVO diagnosis ^a^, n (%)** |  |  |  |  |  |
| Group 1 | 64 (31.1) | 9 (13.9) | 8 (38.1) | 41 (37.6) | 0.054 |
| Group 2 | 74 (35.9) | 27 (41.5) | 6 (28.6) | 37 (33.9) |  |
| Group 3 | 68 (33.0) | 29 (44.6) | 7 (33.3) | 31 (28.5) |  |
| **Treatment duration, day, mean ± SD** | 100.4 ± 77.0 ^d^ | 118.4 ± 89.8 ^d^ | 80.2 ± 51.9 | 111.2 ± 76.4 | 0.375 |
| **Recurrence, n (%)** | 30 (15.8) ^e^ | 9 (14.5) ^e^ | 5 (25.0) | 9 (8.7) | 0.149 |

**Abbreviations:** AD, absolute difference; CV, coefficient of variation; ESR, erythrocyte sedimentation rate; PC, percent change; PVO, pyogenic vertebral osteomyelitis; SD, standard deviation.

1. Group 1: initial-moderate, fast-response; Group 2: initial-high, fast-response; Group 3: initial-high, slow-response.
2. The initial ESR for patients with immediate operation and those with delayed operation was not significantly different (P = 0.196).
3. The ESR-AD for patients with immediate operation and those with delayed operation was not significantly different (P = 0.269).
4. The treatment duration for patients with immediate operation and those with delayed operation was not significantly different (P = 0.097).

The recurrence status for patients with immediate operation and those with delayed operation was not significantly different (P = 0.810).
