# Supplementary material for: First-4-week erythrocyte sedimentation rate variability predicts erythrocyte sedimentation rate trajectories and clinical course among patients with pyogenic vertebral osteomyelitis
Source: PLoS One. 2019 Dec 4;14(12):e0225969. doi: 10.1371/journal.pone.0225969 (PMC6892503; doi:10.1371/journal.pone.0225969)
Supplement: S3 Table — (DOCX) [file pone.0225969.s003.docx]

**S3 Table.** The 4-week erythrocyte sedimentation rate (ESR) variability and 6-month ESR trajectory by the presence of abscess.

| **Variables** | **No abscess**  **(N = 80)** | **Abscess without drainage/operation**  **(N = 75)** | **Abscess with**  **drainage/operation**  **(N = 246)** | **p-value** |
| --- | --- | --- | --- | --- |
| **ESR variability within 4 weeks of PVO diagnosis, mean ± SD** |  |  |  |  |
| Initial ESR | 70.5 ± 29.2 | 77.3 ± 26.2 ^b^ | 76.8 ± 28.3 ^b^ | 0.181 |
| ESR-AD | -11.1 ± 30.5 | -11.4 ± 25.0 | -8.3 ± 30.1 | 0.418 |
| ESR-CV | 25.3 ± 14.8 | 22.5 ± 19.1 | 25.1 ± 18.1 | 0.810 |
| ESR-PC | -5.9 ± 55.4 | -9.7 ± 49.1 | 8.9 ± 189.9 | 0.407 |
| ESR-intercept | 73.9 ± 20.4 | 79.8 ± 19.9 | 77.5 ± 20.8 | 0.382 |
| ESR-Slope | -0.5 ± 0.7 | -0.5 ± 0.6 | -0.4 ± 0.7 | 0.199 |
| **ESR trajectory within 6 months of PVO diagnosis ^a^, n (%)** |  |  |  | 0.114 |
| Group 1 | 33 (41.2) | 25 (33.3) | 64 (26.0) |  |
| Group 2 | 22 (27.5) | 26 (34.7) | 96 (39.0) |  |
| Group 3 | 25 (31.2) | 24 (32.0) | 86 (35.0) |  |
| **Treatment duration, day, mean ± SD** | 100.1 ± 92.7 | 116.3 ± 72.4 ^c^ | 103.5 ± 74.8 ^c^ | 0.986 |
| **Recurrence, n (%)** | 11 (14.7) | 5 (6.8) ^d^ | 37 (16.2) ^d^ | 0.133 |

**Abbreviations:** AD, absolute difference; CV, coefficient of variation; ESR, erythrocyte sedimentation rate; PC, percent change; PVO, pyogenic vertebral osteomyelitis; SD, standard deviation.

1. Group 1: initial-moderate, fast-response; Group 2: initial-high, fast-response; Group 3: initial-high, slow-response.
2. The initial ESR for patients who had abscess without drainage and those who had abscess with drainage was not significantly different (P = 0.918).
3. The treatment duration for patients who had abscess without drainage and those who had abscess with drainage was not significantly different (P = 0.077).
4. The recurrence status for patients who had abscess without drainage and those who had abscess with drainage was significantly different (P = 0.04).
