# Supplementary material for: First-4-week erythrocyte sedimentation rate variability predicts erythrocyte sedimentation rate trajectories and clinical course among patients with pyogenic vertebral osteomyelitis
Source: PLoS One. 2019 Dec 4;14(12):e0225969. doi: 10.1371/journal.pone.0225969 (PMC6892503; doi:10.1371/journal.pone.0225969)
Supplement: S4 Table — (DOCX) [file pone.0225969.s004.docx]

**S4 Table.** Prediction of erythrocyte sedimentation rate (ESR) trajectory group (Groups 1 vs. 2+3) through logistic regression based on ESR within 4 weeks of pyogenic vertebral osteomyelitis diagnosis.

| **Variables** | **Crude OR** (95% CI) | **Model S1-1 (AIC:** **252.1) ^b^** | | **Model S1-2 (AIC: 238.9) ^c^** | | **Model S1-3 (AIC: 240.9) ^d^** | |
| --- | --- | --- | --- | --- | --- | --- | --- |
|  |  | β | Proportional OR (95% CI) | β | Proportional OR (95% CI) | β | Proportional OR (95% CI) |
| Initial ESR, mm/h | 1.05 (1.04, 1.06) | 0.089 | 1.09 (1.07, 1.12) | 0.090 | 1.09 (1.07, 1.12) | 0.090 | 1.09 (1.07, 1.12) |
| ESR **–** AD ≥ −9 mm/h ^a^ | 2.46 (1.56, 3.93) | 3.633 | 37.84 (15.37, 105.48) | 3.682 | 39.72 (14.84, 123.1) | 3.679 | 39.59 (14.76, 122.9) |
| Age, year | 1.02 (1.00, 1.04) |  |  | 0.002 | 1.00 (0.97, 1.03) | 0.003 | 1.00 (0.97, 1.03) |
| Male | 0.65 (0.41, 1.03) |  |  | -0.680 | 0.51 (0.23, 1.10) | -0.683 | 0.51 (0.23, 1.1) |
| Diabetes mellitus | 3.13 (1.91, 5.27) |  |  | 0.686 | 1.99 (0.92, 4.38) | 0.683 | 1.98 (0.92, 4.38) |
| ESRD | 4.78 (1.66, 20.2) |  |  | 0.424 | 1.53 (0.24, 12.85) | 0.413 | 1.51 (0.24, 12.85) |
| Malignancy | 2.82 (1.16, 8.43) |  |  | 1.323 | 3.75 (0.66, 23.21) | 1.318 | 3.74 (0.66, 23.19) |
| eGFR, mL/min/1.73 m² | 0.98 (0.98, 0.99) |  |  | -0.010 | 0.99 (0.97, 1.01) | -0.010 | 0.99 (0.97, 1.01) |
| CCI ≥ 3 | 2.77 (1.56, 5.21) |  |  | -0.224 | 0.80 (0.28, 2.28) | -0.226 | 0.80 (0.28, 2.27) |
| Mixed bacteria | 2.90 (1.10, 10.0) |  |  |  |  | 0.061 | 1.08 (0.23, 5.59) |
| ***C* statistic (95% CI)** |  | **0.91 (0.87 - 0.94)** | | **0.92 (0.89 - 0.95)** | | **0.92 (0.89 - 0.95)** | |

**Abbreviations:** AD, absolute difference; AIC, Akaike Information Criterion; CCI, Charlson’s comorbidities index; CI, confidence interval; eGFR, estimated glomerular filtration rate; ESR, erythrocyte sedimentation rate; ESRD, end-stage renal disease; OR, odds ratio; PVO, pyogenic vertebral osteomyelitis.

1. We used the median value of ESR **–** AD as the cutoff value.
2. In Model 1-1, initial ESR and ESR – AD were included as predicting variables and the ESR groups (1 vs. 2+3) as the dichotomous response variable.
3. In Model 1-2, initial ESR, ESR – AD, demographic information (age, gender), baseline comorbidities (diabetes, ESRD, malignancy, and CCI) and baseline eGFR were included as predicting variables and he ESR groups (1 vs. 2+3) as the dichotomous response variable.
4. In Model 1-3 (full model), initial ESR, ESR – AD, demographic information (age, gender), baseline comorbidities (diabetes, ESRD, malignancy, and CCI), baseline eGFR, and polymicrobial infection were included as predicting variables and the ESR groups (1 vs. 2+3) as the dichotomous response variable.
