# Supplementary material for: First-4-week erythrocyte sedimentation rate variability predicts erythrocyte sedimentation rate trajectories and clinical course among patients with pyogenic vertebral osteomyelitis
Source: PLoS One. 2019 Dec 4;14(12):e0225969. doi: 10.1371/journal.pone.0225969 (PMC6892503; doi:10.1371/journal.pone.0225969)
Supplement: S1 Fig — (DOCX) [file pone.0225969.s005.docx]

**S1 Figure.** Directed acyclic graph (DAG) of the potential causal relationship between covariables and poor prognosis outcomes among patients with PVO.

1. Unadjusted DAG

**
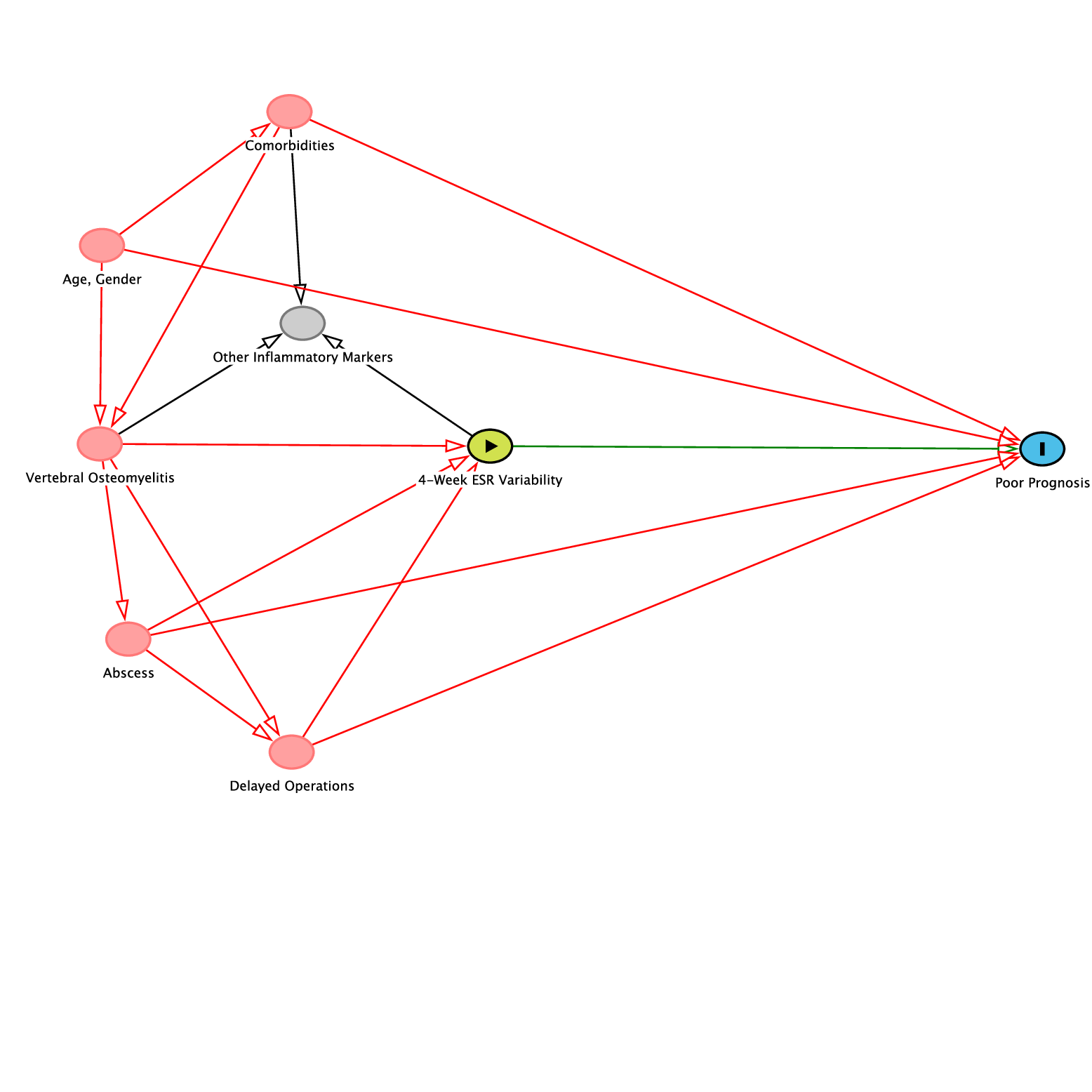
**

1. DAG adjusted for age, gender, comorbidities (i.e., diabetes, ESRD, malignancy, Charlson’s Comorbidity Index), other inflammatory marker (i.e., CRP), the presence of abscess, and delayed operation.

**
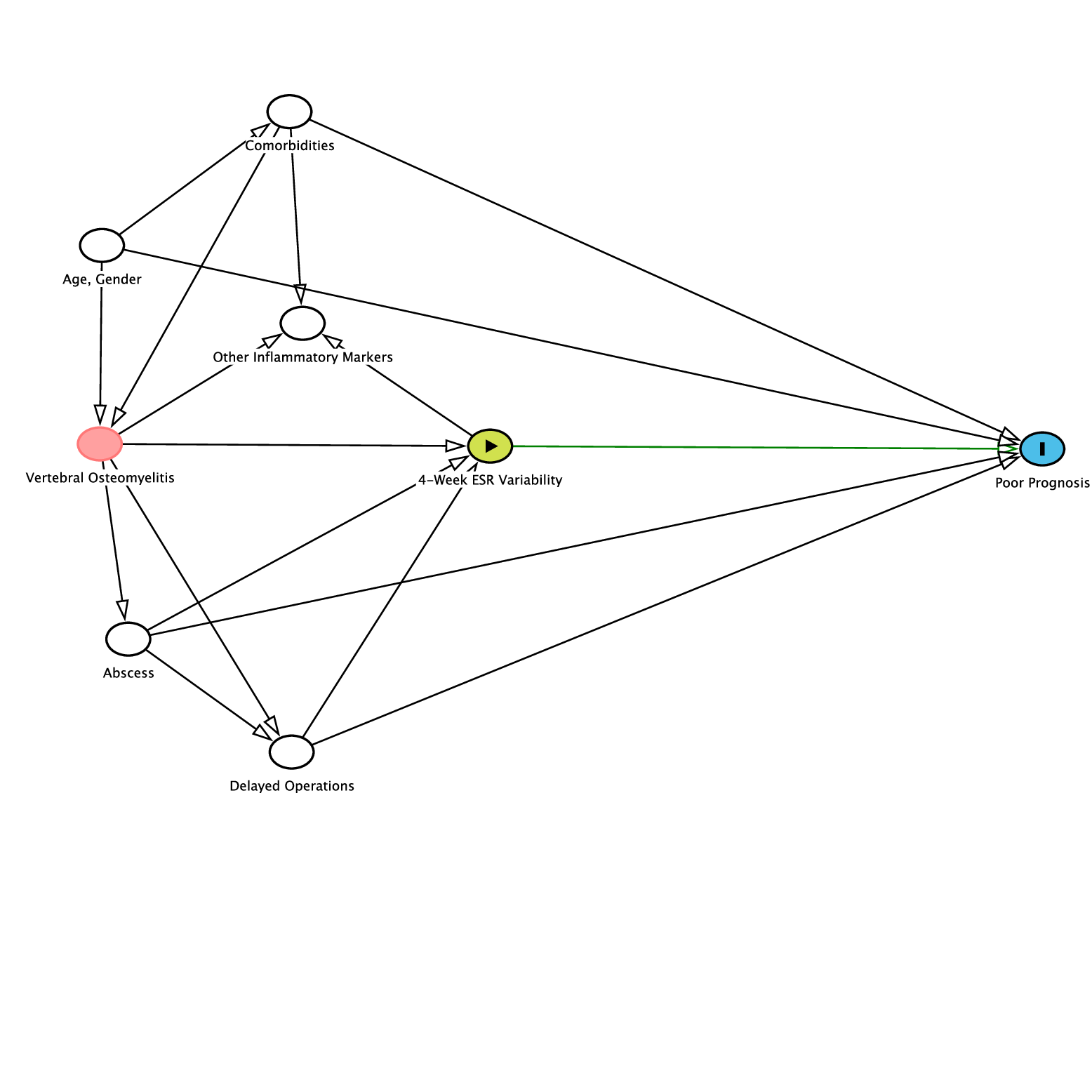
**
