# Supplementary material for: First-4-week erythrocyte sedimentation rate variability predicts erythrocyte sedimentation rate trajectories and clinical course among patients with pyogenic vertebral osteomyelitis
Source: PLoS One. 2019 Dec 4;14(12):e0225969. doi: 10.1371/journal.pone.0225969 (PMC6892503; doi:10.1371/journal.pone.0225969)

**S2 Figure.** Group-based trajectory modeling of 6-month C-reactive protein (CRP) and 6-month white blood cell count (WBC) among patients with pyogenic vertebral osteomyelitis.

(A) CRP (B) WBC


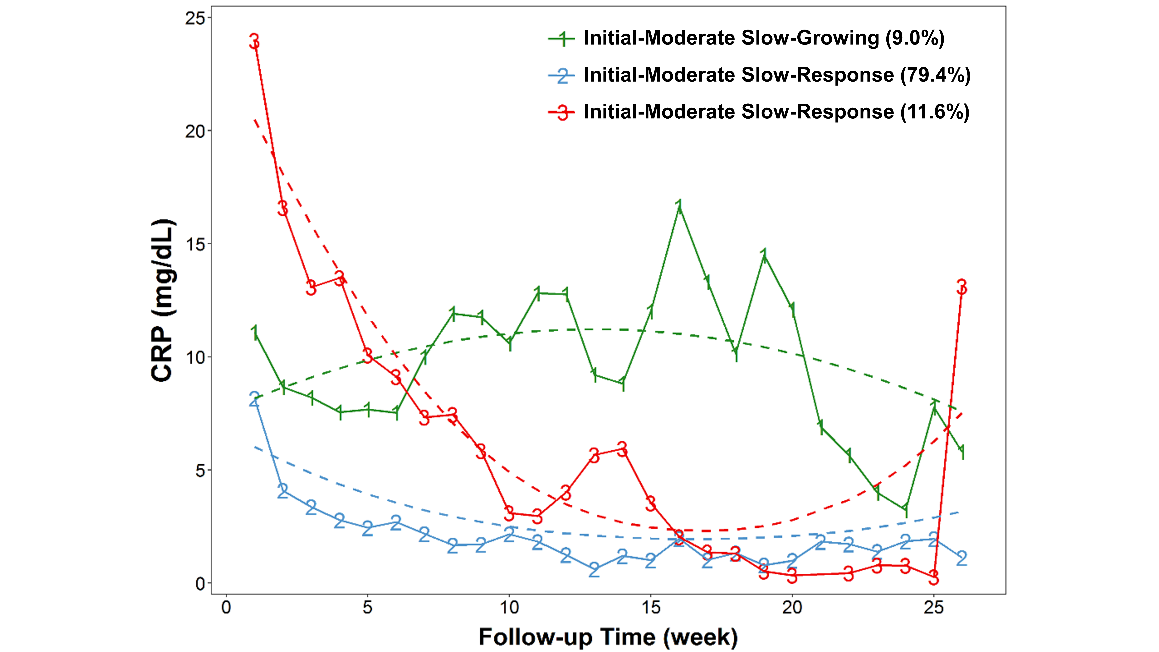

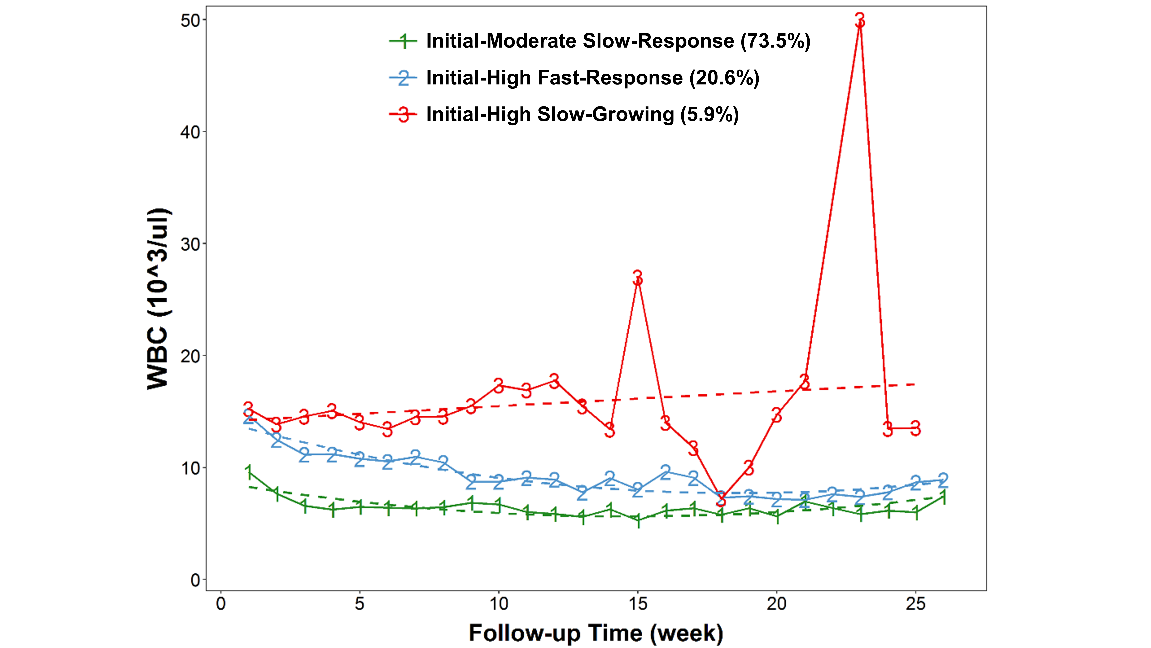

Supplement: S2 Fig — (DOCX) [file pone.0225969.s006.docx]
