# Supplementary material for: First-4-week erythrocyte sedimentation rate variability predicts erythrocyte sedimentation rate trajectories and clinical course among patients with pyogenic vertebral osteomyelitis
Source: PLoS One. 2019 Dec 4;14(12):e0225969. doi: 10.1371/journal.pone.0225969 (PMC6892503; doi:10.1371/journal.pone.0225969)
Supplement: S3 Fig — (DOCX) [file pone.0225969.s007.docx]

**S3 Figure.** Receiver operating characteristic curve for the predictive model of 6-month recurrence.


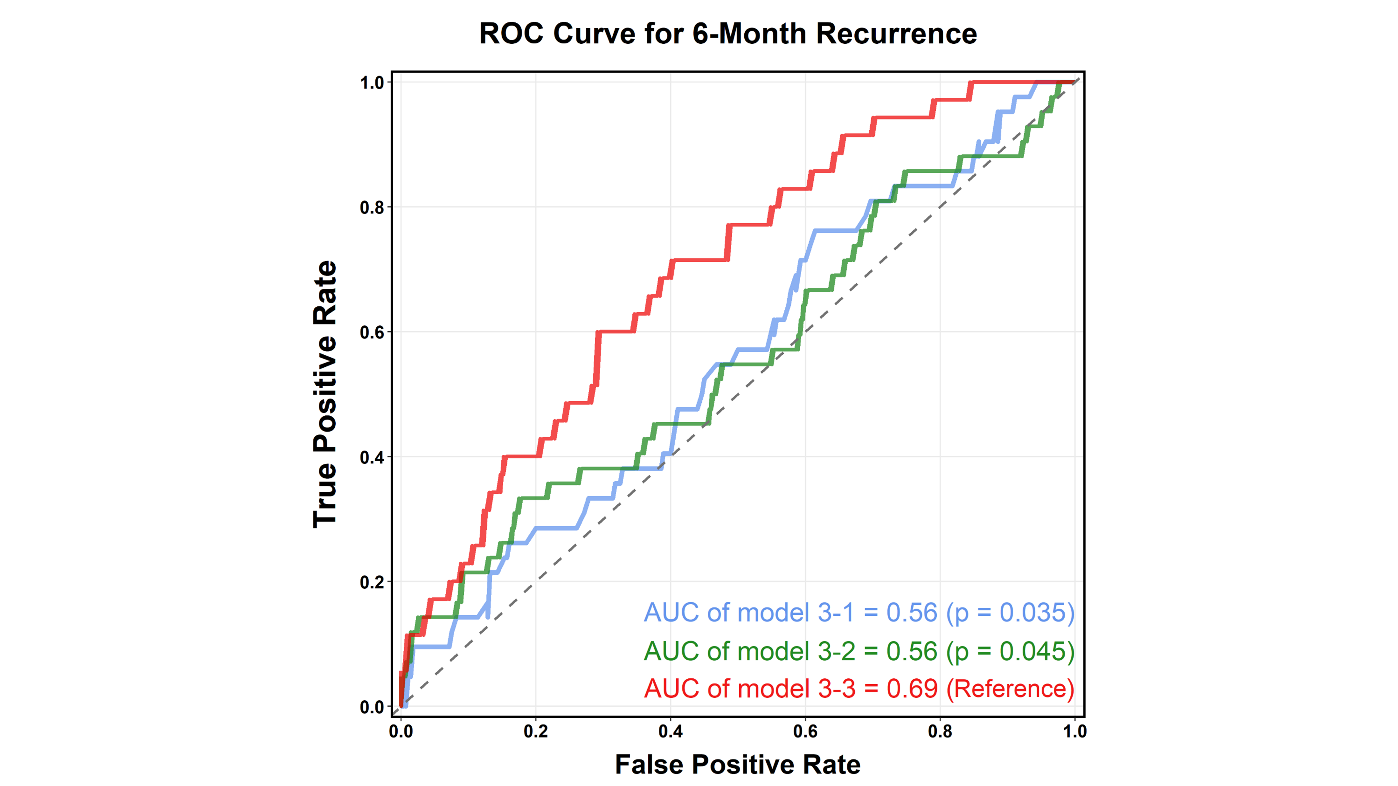


**Footnotes:**

Predictors in Model 3-1: Initial ESR, ESR − AD ≥−9.

Predictors in Model 3-2: Initial ESR, ESR − AD ≥−9, ESR − CV.

Predictors in Model 3-3: Initial ESR, ESR − AD ≥−9, ESR − CV, initial CRP, age, gender, diabetes, ESRD, malignancy, abscess, delayed operation, Charlson’s comorbidities index ≥ 3.

**Abbreviations:** AD, absolute difference; AUC, area under the ROC curve; CRP, C-reactive protein; CV, coefficient of variation; ESR, erythrocyte sedimentation rate; ESRD, end-stage renal disease; PVO, pyogenic vertebral osteomyelitis; ROC, receiver operating characteristic curve.
