# Supplementary material for: First-4-week erythrocyte sedimentation rate variability predicts erythrocyte sedimentation rate trajectories and clinical course among patients with pyogenic vertebral osteomyelitis
Source: PLoS One. 2019 Dec 4;14(12):e0225969. doi: 10.1371/journal.pone.0225969 (PMC6892503; doi:10.1371/journal.pone.0225969)
Supplement: S5 Fig — (DOCX) [file pone.0225969.s009.docx]

**S5 Figure.** Calibration plot for the full predictive model of 6-month recurrence (Model 3-3).


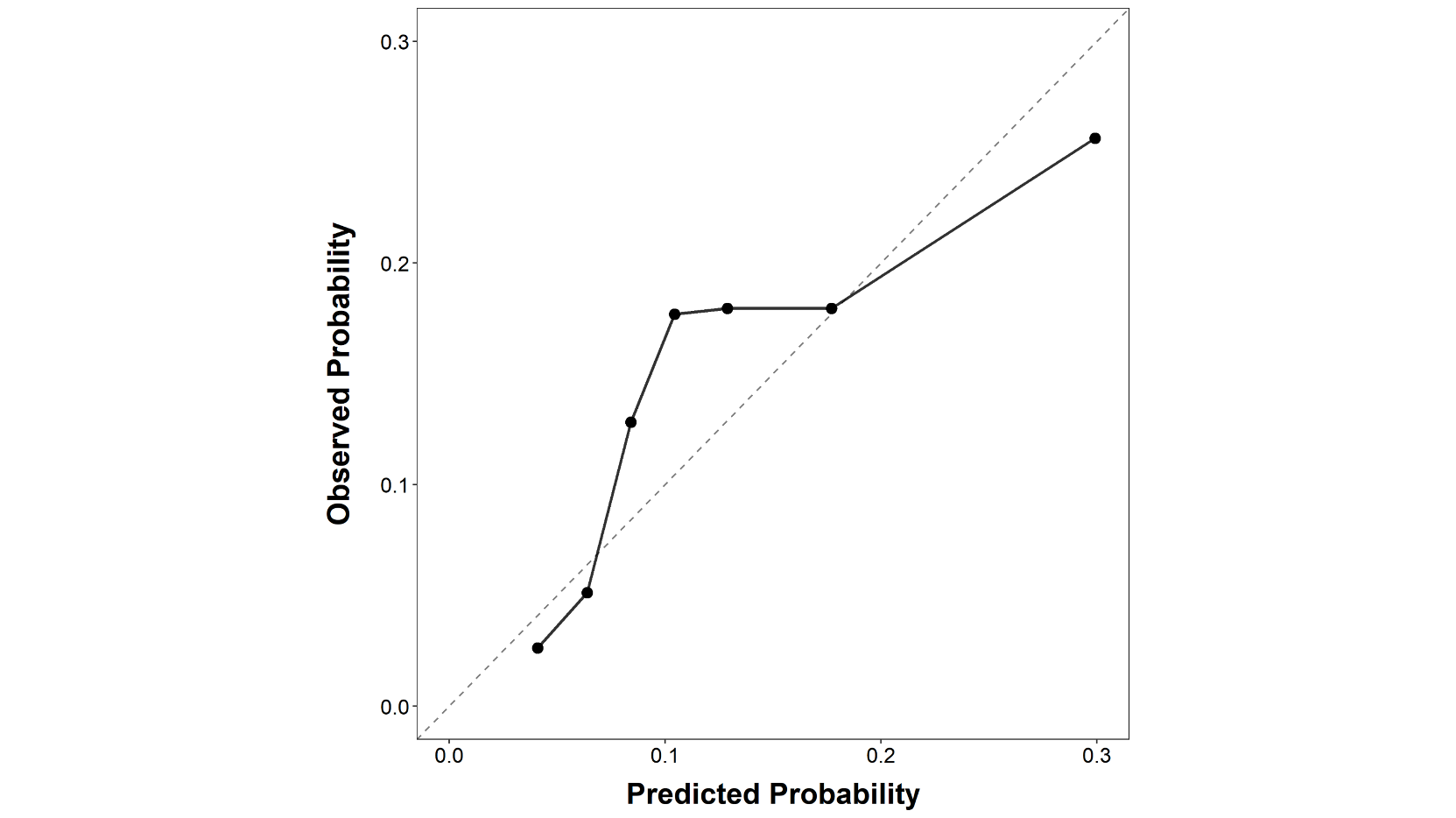


**Footnotes:**

The *P*-value from the Hosmer-Lemeshow Goodness-of-fit test was 0.75.
